# Mental health outcomes in sexual violence victims: Age group differences and trauma-related risk factors in digital media-related cases

**DOI:** 10.1192/j.eurpsy.2026.12002

**Published:** 2026-07-31

**Authors:** S. Lee, M. Hong, W. S. Kang

**Affiliations:** 1 Psychiatry, Kyung Hee University Hospital at Gangdong; 2 Psychiatry, Kyung Hee University Hospital; 3 Psychiatry, Kyung Hee University College of Medicine, Seoul, Korea, Republic Of

## Abstract

**Introduction:**

Sexual violence mediated through digital media, including videos, photographs, and online communication, poses unique and escalating risks for victims’ mental health. While depressive symptoms are common consequences, little is known about how these outcomes differ by age group and trauma-related factors in digital media–related cases.

**Objectives:**

This study aimed to investigate age group differences and trauma-related risk factors associated with depressive outcomes among sexual violence victims, focusing specifically on cases involving digital media.

**Methods:**

We analyzed 84 victims of digital media–related sexual violence who visited a support center. Participants were categorized into two groups based on depressive symptom scores: low (n=55) and high (n=29). Sociodemographic variables, crime-related circumstances, perpetrator characteristics, and motives for visiting the center were compared using chi-square tests.

**Results:**

All participants were victims of sexual violence involving digital media. Significant differences emerged across several domains:

Age group: Adults were more likely to present with high depressive scores than adolescents (60.6% vs. 17.7%, *p*<.0001).

Crime circumstances: Within this digital media–related sample, cases involving alcohol use or loss of consciousness were strongly associated with higher depressive scores (83.3%), whereas videos/photography cases showed lower depressive scores by comparison (*p*=0.033).

Perpetrator age: Victims assaulted by perpetrators aged 20–30 years exhibited higher depressive scores (57.1%, *p*=0.021).

Help-seeking motives: Voluntary visitors more often reported high depressive scores than involuntary ones (53.9% vs. 25.9%, *p*=0.013).

Other factors, including sex, psychiatric history, number of perpetrators, and past victimization, were not significantly different.

**Image 1:**
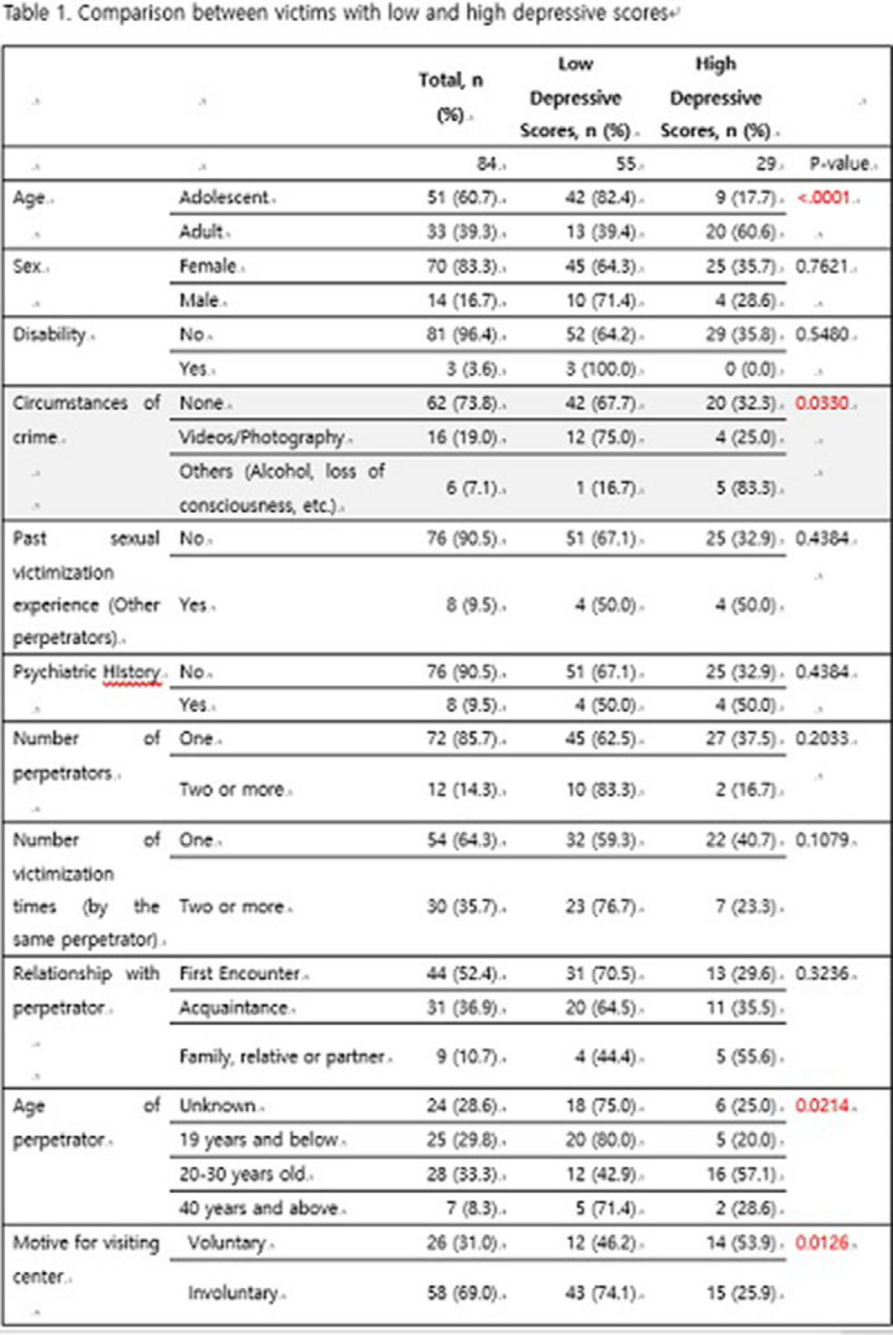
Image 1: Long description.

**Conclusions:**

Among victims of digital media–related sexual violence, mental health outcomes varied according to age group, trauma circumstances, and perpetrator characteristics. Adolescents were more likely to report lower depressive scores, potentially reflecting delayed symptom expression, while adults exhibited greater immediate distress. Importantly, cases involving alcohol or impaired consciousness carried the highest depressive burden, whereas video/photography cases displayed a different pattern of symptom severity. These age-related differences underscore the need for distinct, age-sensitive intervention strategies: proactive monitoring and long-term follow-up for adolescents, and immediate intensive support for adults.

Acknowledgement: Preliminary findings from this dataset were presented at the APA Annual Meeting 2025; the present abstract reflects extended analyses with a focus on age group differences and trauma-related risk factors.

**Disclosure of Interest:**

None Declared

## Long descriptions

### Image 1: Long description

Table 1 titled Comparison between victims with low and high depressive scores. The table has four main columns: Total, n (%); Low Depressive Scores, n (%); High Depressive Scores, n (%); and P-value. The total sample size is 84, with 55 in the low group and 29 in the high group.

* Age: Adolescent (51 total, 60.7%) vs Adult (33 total, 39.3%). P-value < .0001.

* Sex: Female (70 total, 83.3%) vs Male (14 total, 16.7%). P-value 0.7621.

* Disability: No (81 total, 96.4%) vs Yes (3 total, 3.6%). P-value 0.5480.

* Circumstances of crime: None (62 total, 73.8%), Videos/Photography (16 total, 19.0%), Others (6 total, 7.1%). P-value 0.0330.

* Past sexual victimization experience: No (76 total, 90.5%) vs Yes (8 total, 9.5%). P-value 0.4384.

* Psychiatric History: No (76 total, 90.5%) vs Yes (8 total, 9.5%). P-value 0.4384.

* Number of perpetrators: One (72 total, 85.7%) vs Two or more (12 total, 14.3%). P-value 0.2033.

* Number of victimization times: One (54 total, 64.3%) vs Two or more (30 total, 35.7%). P-value 0.1079.

* Relationship with perpetrator: First Encounter (44 total, 52.4%), Acquaintance (31 total, 36.9%), Family, relative or partner (9 total, 10.7%). P-value 0.3236.

* Age of perpetrator: Unknown (24 total, 28.6%), 19 years and below (25 total, 29.8%), 20-30 years old (28 total, 33.3%), 40 years and above (7 total, 8.3%). P-value 0.0214.

* Motive for visiting center: Voluntary (26 total, 31.0%) vs Involuntary (58 total, 69.0%). P-value 0.0126.

Significant P-values (less than 0.05) are highlighted in red text for Age, Circumstances of crime, Age of perpetrator, and Motive for visiting center.

Navigate back to Image 1:.

